# Case of a Woman Who, by the Means of Galvano-Puncture, Recovered Her Speech, after Having Lost It for Twenty-three Years

**Published:** 1848-05

**Authors:** 


*Case of a Woman who, by the means of Galvano-Puncture, recovered her speech after having lost it for twenty-three years.* By M. Camino.—In 1813, a married woman, Rosa Ponti, then aged 47, was affected with a loss of sensation and movement, in consequence of a great fright. Recovering by little and little, she regained the use of her legs, but did not recover that of her arms and head, which remained paralysed from that time. From that moment she could not articulate a single word; she stammered sometimes, but without being able to pronounce even a monosyllable. The tongue, which remained immoveable between the teeth, appeared also to be atrophied.

On the 21st of May, 1836, a metallic needle was introduced into the neck, directing its point towards the occipital branch of the first cervical nerve; then it was brought into connexion with the zinc pole of a voltaic pile; and holding the tongue, elevated and stretched out on a sheet of the same metal, the circle was closed by presenting to that organ the knob of a brass director. The patient showed, by quickly drawing herself away, that she had felt the shock. The experiment was repeated, and the effect was more marked than before. She gained immediately the power of lifting her tongue. At the end of three other shocks the patient exclaimed—"Oh, Dieu!" and could answer some questions in an intelligent manner, although with some difficulty. She also became able to move her tongue from side to side.

The next day, after some shocks given in the same manner, M. Camino commenced to vary the points of contact, and to give the electricity different directions. The patient showed more and more sensibility, and the faculty of articulation followed the gradual return of the movement of the tongue.

Two days of repose employed in exercising the organ sensibly rendered the faculty of pronouncing and articulating sounds easier and more accurate. In a short time she was able to speak as before, and acquitted herself so well, and with so much ardour, that she seemed, says the author, to wish to make up as quickly as possible for the time lost in inaction and silence.

Every three or four days she came back to receive four or five shocks with the pile, not being able, as she says, to bear more.

On the 10th of June, she complained, without obvious cause, of pain in the head and a general feeling of weight, an ailment which was dissipated by a bleeding.

After some more sittings, not only was her speech recovered, but also the activity of the other paralysed parts, which became quite fit to exercise their functions.—*Gaz. Med. de Paris.*

